# Postoperative secondary contralateral arachnoid cyst following rupture and bleeding of intracranial aneurysm: a case report

**DOI:** 10.3389/fsurg.2025.1672623

**Published:** 2025-09-23

**Authors:** Kun Hu, Yanyan Yu, Yancong Yang, Huichen Li, Jinyuan Liao, Huanglian Zhong, Qiuhua Jiang, Jun Liu, Shuiying Zeng, Nan Yang, Wenjun Zhang

**Affiliations:** ^1^ Department of Neurosurgery, The Ganzhou Affiliated Hospital, Jiangxi Medical College, Nanchang University, Ganzhou, Jiangxi, China; ^2^ Department of Neurosurgery, The Second Affiliated Hospital, Jiangxi Medical College, Nanchang University, Nanchang, Jiangxi, China; ^3^ Department of Rehabilitation Medicine, The Ganzhou Affiliated Hospital, Jiangxi Medical College, Nanchang University, Ganzhou, Jiangxi, China

**Keywords:** intracranial aneurysm, arachnoid cyst, surgery, endoscopic techniques, diagnosis

## Abstract

The simultaneous occurrence of intracranial aneurysms and intracranial arachnoid cysts is a rare clinical observation, with the majority of documented instances demonstrating ipsilateral presentation. In this report, we describe an atypical case involving the development of a secondary arachnoid cyst subsequent to the rupture of an intracranial aneurysm. Notably the cyst was situated contralaterally to the site of the aneurysm rupture and outside the surgical field. The patient's clinical history and imaging studies confirmedcorroborated the secondary nature of the cyst, which is postulated to have resulted from inflammatory responses triggered by a subarachnoid hemorrhage (SAH). The patient underwent neuroendoscopic partial resection of the cyst wall and lateral ventriculostomy, leading to a significant improvement in neurological dysfunction symptoms associated with the secondary arachnoid cyst. Follow-up cranial MRI demonstrated a substantial reduction in the cyst's volume, with no evidence of subsequent hydrocephalus or cyst enlargement. This case enhances the comprehension of the pathophysiological mechanisms underlying the formation of contralateral arachnoid cysts subsequent to aneurysm rupture and emphasizes the necessity of acknowledging arachnoid cysts as potential delayed complications associated with aneurysmal subarachnoid hemorrhage (aSAH).

## Introduction

Intracranial aneurysms and intracranial arachnoid cysts are common conditions encountered in neurosurgical practice; nevertheless, their concurrent manifestation in a single patient is an infrequent phenomenon. In most documented instances, the arachnoid cyst is identified concurrently with the intracranial aneurysm during imaging procedures, with almost all previously reported cases exhibiting showing ipsilateral localization of both pathologies (1). The occurrence of an arachnoid cyst following surgical intervention for a ruptured cerebral aneurysm is rare, and the formation of a contralateral cyst distant from the ipsilateral surgical site, has not been reported in the literature (2–4). This study introduces a unique case of a contralateral arachnoid cyst emerging postoperatively after the rupture of an intracranial aneurysm and investigates the possible mechanisms responsible for contralateral cyst formation, potentially marking the first documented occurrence. Furthermore, it is crucial for clinicians to acknowledge that arachnoid cysts may represent delayed complications of aneurysmal subarachnoid hemorrhage (aSAH).

## Case report

**Figure 1:**
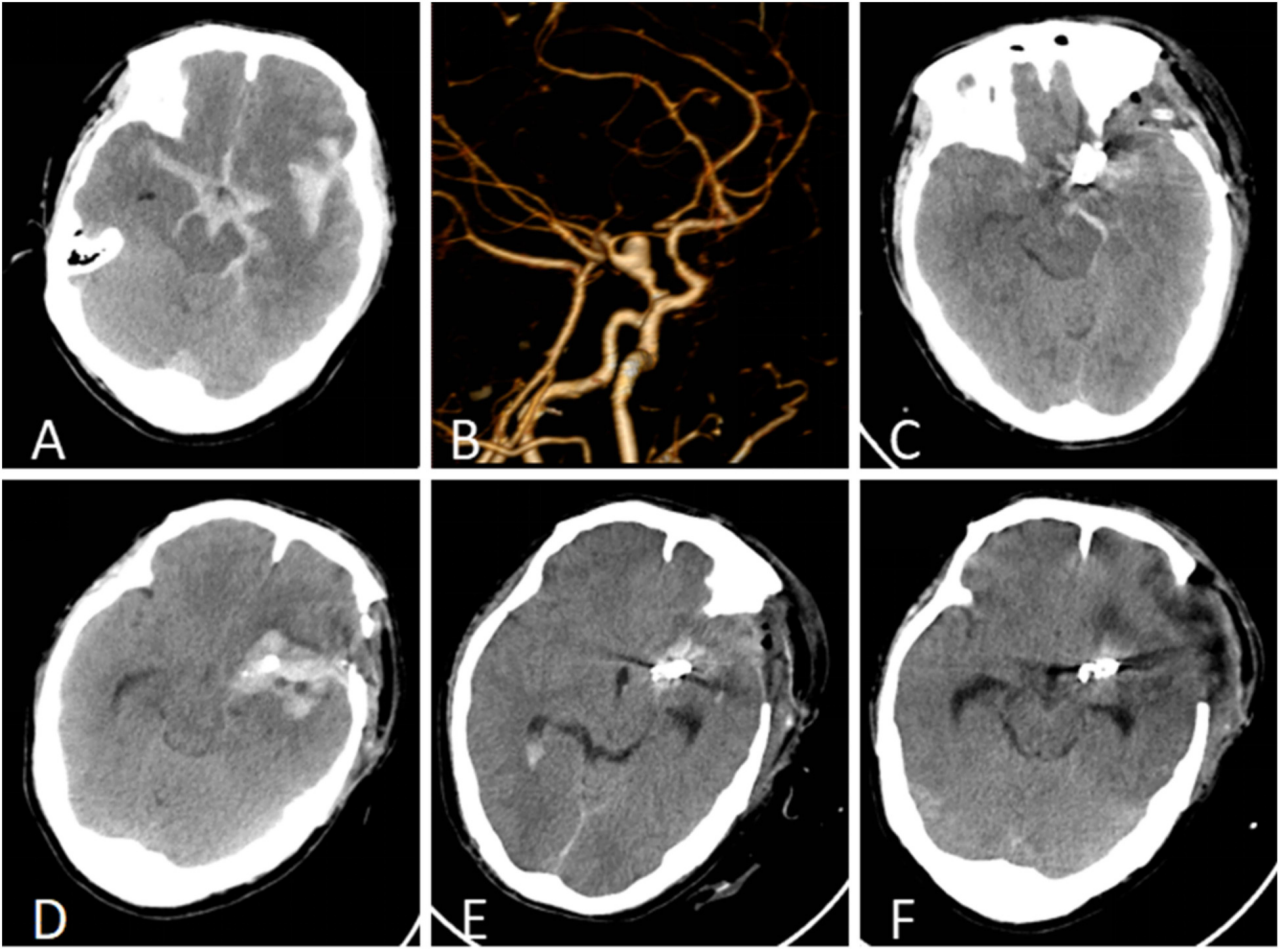
**(A)** Cranial CT identified a subarachnoid hemorrhage and a hematoma within the left fissure cistern. **(B)** CTA revealed an aneurysm in the ophthalmic segment of the left internal carotid artery. **(C)** The initial postoperative CT follow-up indicated a reduction in intracranial hemorrhage following cerebral aneurysm clipping. **(D)** Rebleeding was detected 10 days post-discharge, with an emergent cranial CT scan revealing a hematoma in the left frontotemporal region. **(E)** The initial follow-up CT after the second surgical intervention confirmed successful clipping of the cerebral aneurysm and substantial resolution of the temporal hematoma. **(F)** At the time of discharge, postoperative cranial CT demonstrated postsurgical changes without signs of hydrocephalus or cyst formation.

**Figure 2:**
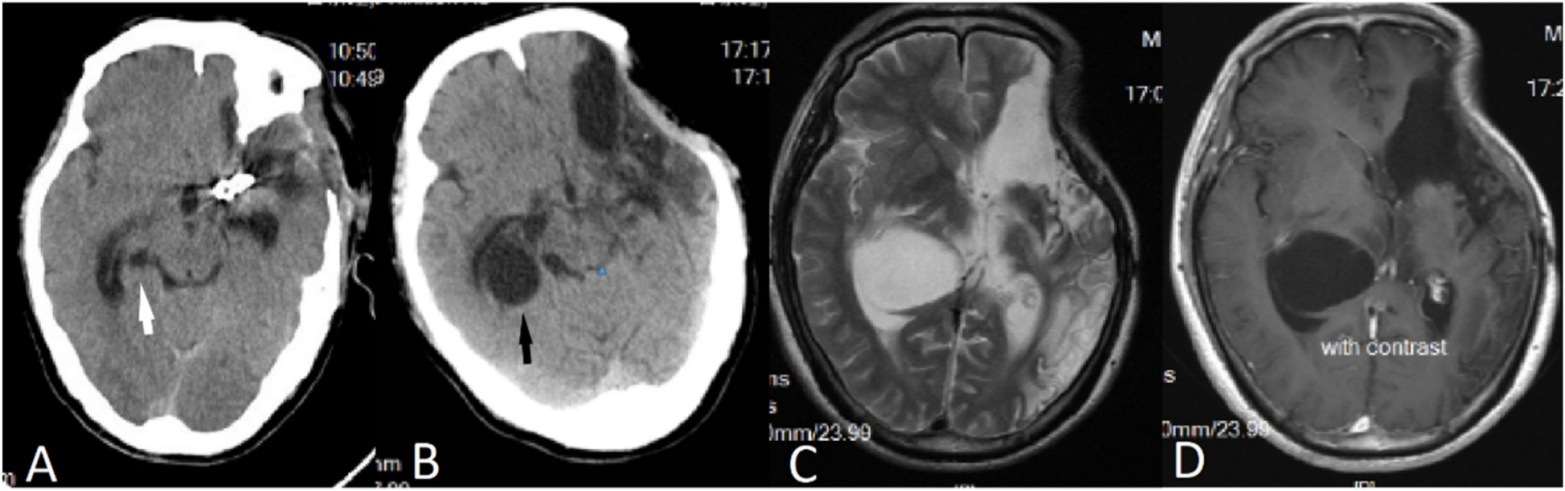
**(A)** Fifteen days post-surgery, a CT scan revealed the presence of an arachnoid cyst located dorsal to the right ambient cistern and medial to the parahippocampal gyrus (indicated by a white arrow). **(B)** A follow-up CT scan conducted three months later demonstrated an enlargement of the arachnoid cyst (indicated by a black arrow). (**C,D**) Subsequent MRI findings indicated that the cystic fluid exhibited a signal identical to that of CSF, with no enhancement observed on the contrast-enhanced scan.

**Figure 3:**
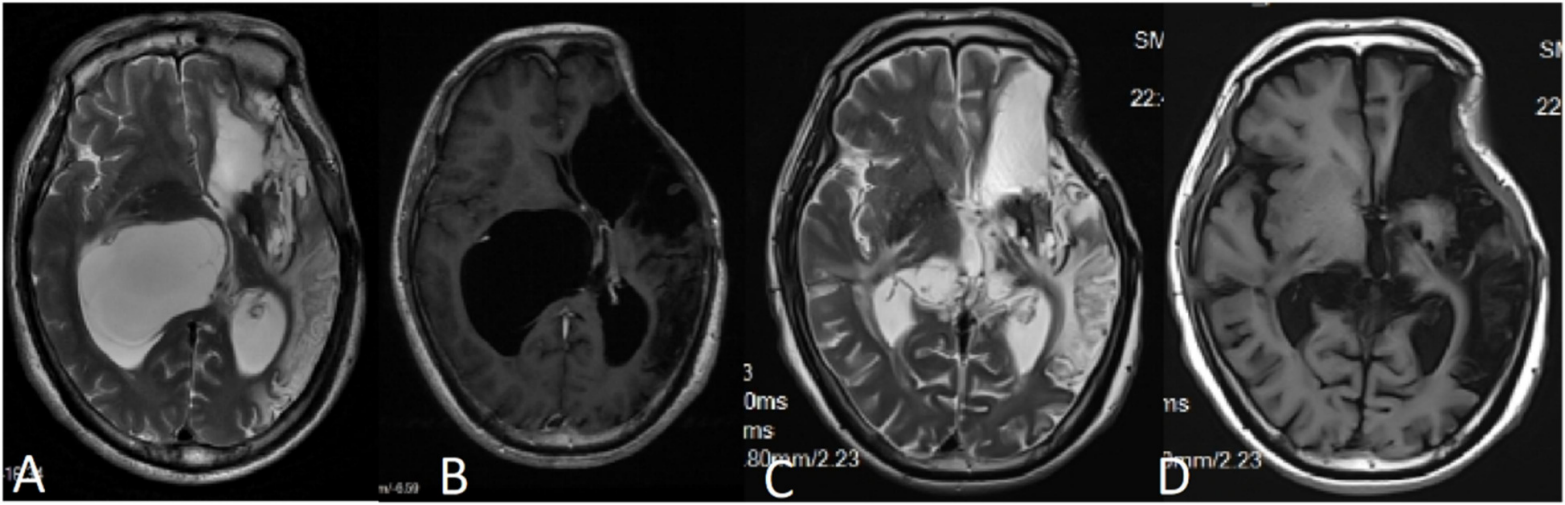
**(A,B)** MRI conducted 12 months post-craniotomy revealed a further enlargement of the arachnoid cyst accompanied by a significant mass effect, resulting in noticeable compression of the thalamus. (C,D) Six months following the partial resection of the cyst wall, MRI findings indicated a reduction in the mass effect exerted by the cyst, with no evidence of recurrence.

**Figure 4:**
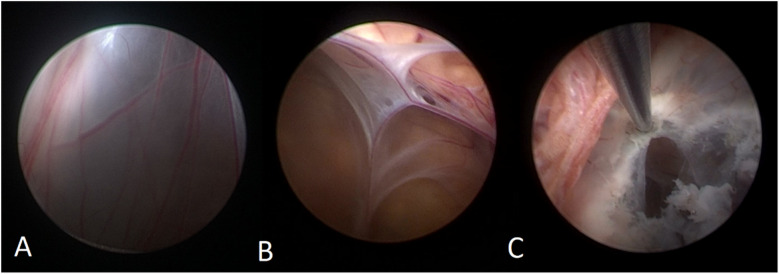
**(A)** The neuroendoscopic image illustrates vascular hyperplasia within the cyst wall, accompanied by tension in the cyst wall. **(B)** The internal perspective reveals that the cyst is closely adherent to the cerebral ventricular wall, with blood vessels situated between the arachnoid membranes. **(C)** The thick-walled cyst is positioned adjacent to the choroid plexus, and it exhibited a reduction in size following cauterization of the cyst wall.

**Figure 5:**
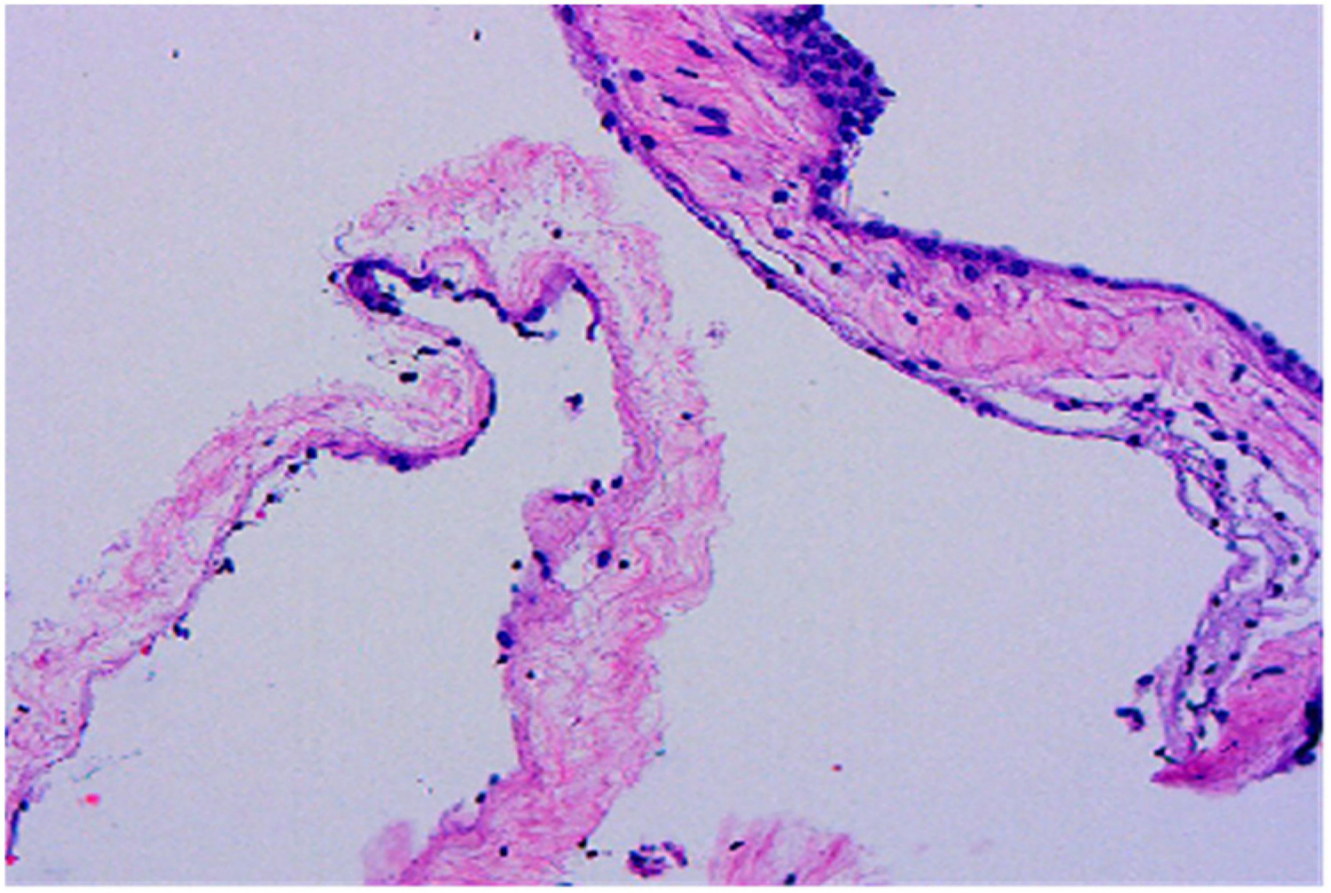
Histopathological examination reveals cyst walls composed of proliferative fibrous tissue lined with cuboidal or flattened epithelial cells (HE × 100).

A 50-year-old male patient was admitted with a complaint of a persistent headache lasting for three hours. His medical history was notable for a 20-year history of smoking, with no evidence of hypertension or diabetes. Upon physical examination, the patient was found to be alert and oriented, with a Glasgow Coma Scale (GCS) score of 15 (E4V5M6). The pupils were bilaterally isocoric at 3.0 mm and exhibited intact light reflexes. Signs of meningeal irritation were absent, and no significant neurological deficits were detected. Cranial computed tomography (CT) and computed tomography angiography (CTA) revealed a subarachnoid hemorrhage and an aneurysm of the left internal carotid artery at the ophthalmic segment (Figures 1A,B). He was classified as Hunt-Hess grade II and modified Fisher grade III. The patient underwent a craniotomy for aneurysm clipping. The initial postoperative CT scan of the head demonstrated post-clipping alterations and a marked reduction in the intracranial hematoma relative to prior imaging (Figure 1C). Following this, the patient was administered continuous lumbar drainage of hemorrhagic cerebrospinal fluid (CSF) in conjunction with appropriate supportive care. Three weeks postoperatively, the patient demonstrated a favorable recovery and was discharged without complications. However, 10 days after discharge, the patient was readmitted in a state of sudden coma. The left pupil was noted to be dilated with an absent light reflex. An emergency cranial CT scan revealed the formation of a hematoma in the left frontotemporal lobe, indicating the possibility of rebleeding at the surgical site (Figure 1D), the patient underwent an emergency craniotomy for the evacuation of a hematoma. Intraoperative exploration revealed a recurrent and reruptured aneurysm located in the ophthalmic segment. Successful aneurysm clipping was achieved, along with hematoma evacuation and decompressive craniectomy (Figure 1E). The procedure was completed without complications. After an uncomplicated clinical recovery, the patient was discharged. The pre-discharge follow-up cranial CT scan showed postoperative changes in the brain, with no other significant abnormal findings observed (Figure 1F). Fifteen days post-discharge, the patient exhibited cognitive decline and left-sided weakness. A readmission cranial CT scan revealed a cystic lesion located dorsal to the right ambiens cistern and medial to the parahippocampal gyrus (Figure 2A). Serial imaging conducted at a three-month interval demonstrated progressive enlargement of the cystic lesion (Figure 2B). Subsequent cranial magnetic resonance imaging (MRI) excluded the presence of gliomas and cholesteatomas (Figures 2C,D). One year later, amidst ongoing neurological deterioration, follow-up cranial CT indicated further expansion of the cystic lesion, accompanied by a significant mass effect (Figures 3A,B). Neuroendoscopic exploration was conducted under general anesthesia, during which a cortical fenestration of the temporal lobe was created to facilitate access to the cystic cavity. Intraoperative observations revealed a thickened cyst wall under high tension with evidence of vascular proliferation. The cystic fluid was colorless and transparent, lacking hemosiderin deposition (Figures 4A–C). An arachnoid cyst was suspected intraoperatively, prompting partial resection of the cyst wall and the creation of a lateral ventriculostomy. The fistulous opening measured approximately 2.5 cm in diameter, facilitating communication with the lateral ventricle (Figure 4C). Histopathological analysis of the cyst wall confirmed the diagnosis of an arachnoid cyst (Figure 5). Following the surgical procedure, the patient demonstrated a gradual enhancement in left-sided motor weakness and cognitive abilities. At the three-month follow-up, both motor and cognitive functions had nearly returned to baseline levels. The GCS score was recorded at 15 (E4V5M6). The latest cranial MRI revealed a substantial reduction in cyst volume, accompanied by a complete resolution of the mass effect (Figures 3C,D).

## Discussion

Intracranial aneurysms and arachnoid cysts are relatively prevalent anomalies observed in neurosurgical practice. Intracranial aneurysms are found in approximately 3%–5% of the general population (5), whereas arachnoid cysts constitute about 1% of all intracranial space-occupying lesions (6). Despite their individual prevalence, the concurrent manifestation of both conditions in a single patient is uncommon (1, 2, 7). In most documented instances, arachnoid cysts are frequently identified alongside intracranial aneurysms during imaging studies. Aneurysms are predominantly located at the bifurcation of the middle cerebral artery or in the region of the carotid-posterior communicating artery, whereas arachnoid cysts are primarily situated in the middle cranial fossa. Importantly, the aneurysms are consistently positioned adjacent to or within these cysts (1). The anatomical colocalization within the subarachnoid space is a fundamental prerequisite for their coexistence. Moreover, the presence of an aneurysm can alter local hemodynamics, subsequently affecting CSF dynamics and pressure gradients within the subarachnoid space, potentially creating conditions favorable for the development of arachnoid cysts. However, the formation of arachnoid cysts secondary to ruptured intracranial aneurysms, particularly those occurring contralaterally at non-surgical sites, has not been documented in the existing literature. This case may represent the first recorded instance of this rare phenomenon. The precise pathogenesis underlying the concurrent development of intracranial aneurysms and arachnoid cysts remains poorly understood. Current evidence suggests that these lesions may be either primary (congenital), involving genetic factors (8, 9) or developmental anomalies (10), or secondary, typically induced by hemorrhage, infection, or trauma (11, 12). Congenital variants demonstrate a strong association with inheritable connective tissue disorders. In this regard, autosomal dominant polycystic kidney disease (ADPKD) is notably linked to both intracranial aneurysms and arachnoid cysts. Schievink reports that the prevalence of intracranial aneurysms in individuals with ADPKD is 10.8%, whereas the prevalence of arachnoid cysts is 8.1% (8). In the current case, the absence of a history of ADPKD, the lack of extracranial anomalies on preoperative imaging, and a negative family history collectively rule out the diagnosis of autosomal dominant polycystic kidney disease. Given the absence of intracranial cystic lesions preoperatively and the clinical presentation, it is postulated the arachnoid cyst is secondary, potentially associated with subarachnoid hemorrhage following the rupture of an intracranial aneurysm.

Current evidence indicates that the development of secondary arachnoid cysts may be linked to aseptic inflammatory responses at the leptomeningeal-arachnoid interface, triggered by subarachnoid hemorrhage. Postprocedural arachnoiditis commonly occurs following aneurysm clipping or coil embolization, with its chronic inflammatory process identified as a key factor in cystogenesis (13, 14). Studies suggest that recurrent subarachnoid hemorrhage and occult meningitis, resulting from lumbar drainage, may exacerbate arachnoiditis development (15, 16). Additionally, prolonged bed rest and the use of intraoperative fibrin sealants are acknowledged as risk factors for adhesive arachnoiditis (17). Although surgical intervention, trauma, and infection are well-known etiological factors for adhesive arachnoiditis, growing evidence suggests that blood products within the subarachnoid space-particularly erythrocyte degradation products-substantially increase the severity of arachnoiditis, leading to arachnoidal fibrosis, hyperplasia, and obliteration of the subarachnoid space (18–21). This inflammatory cascade has the potential to alter CSF dynamics, resulting in flow obstruction that ultimately facilitates cystogenesis. In the current case, the arachnoid cyst developed on the side opposite to the ruptured aneurysm, yet it was located at a distance from the ipsilateral surgical site. This unusual localization may be associated with the patient's postoperative decubitus position, which favored the contralateral side. This positioning could have promoted the gravitational accumulation of blood products in dependent regions, thereby creating conditions conducive tocyst formation. The precise mechanisms underlying the enlargement of arachnoid cysts remain incompletely understood. Several theories have been proposed, including the ball-valve mechanism (22), active fluid secretion by the cyst walls (23), and osmotic gradients between the cyst contents and the CSF (24).

A neuroendoscopic evaluation of the arachnoid cyst revealed a bilaminar arachnoid wall characterized by intervening vascular proliferation. The cyst maintained its structural integrity under tension and was situated adjacent to the choroid plexus and the medial wall of the lateral ventricle, with no observable slit-like formations. The cystic fluid was colorless and transparent, resembling CSF, and comparative analysis confirmed its biochemical similarity to CSF. Immunohistochemical analysis demonstrated immunopositivity for epithelial membrane antigen (EMA). Definitive histological examination confirmed a fibrotic cyst wall devoid of epithelial cellular components. In this case, we propose that cyst enlargement may not primarily involve ball-valve mechanisms or osmotic gradients. Instead, it is plausible that unrestricted communication between the cyst and the subarachnoid space facilitates the transmission of CSF pulsations into the cyst cavity, or that active fluid secretion by the cyst wall itself may contribute to the expansion. Additionally, intraoperative observations suggest that the pronounced vascular proliferation within the cyst wall may expedite this enlargement process.

Arachnoid cysts are typical benign entities, with clinical presentations that vary depending on their anatomical location and size. These cysts may result in increased intracranial pressure, seizures, cerebral compression, and localized mass effects, including cognitive decline, cerebellar ataxia, and hemiparesis. Instances of spontaneous regression have been documented in isolated case reports. Currently, there is no standardized management protocol for arachnoid cysts. Common surgical interventions include cystoperitoneal shunting, cyst fenestration, and complete or partial cystectomy (25). Shunt procedures carry risks such as infection and shunt obstruction, while fenestration is prone to recurrence. Although complete cystectomy is theoretically the most effective strategy, the presence of dense adhesions between the cyst walls and normal neurovascular structures often renders total resection unfeasible. Given the secondary nature and contralateral non-surgical location of this rare cyst, we preformed a neuroendoscopic partial cystectomy alongside a ventriculostomy. This approach led to: (1) effective alleviation of the mass effect; (2) restoration of patency in the proximal cistern and Sylvian fissure; (3) reestablishment of construction of CSF circulation; and (4) symptomatic relief with a diminished risk of recurrence. The postoperative resolution of cyst-induced neurological deficits highlights the minimally invasive nature, favorable outcomes, and low complication rate associated with neuroendoscopic management.

Beyond the well-documented acute complications of aSAH, such as cerebral vasospasm, hydrocephalus, and rebleeding, clinical evidence suggests that the delayed onset of arachnoid cysts may constitute a rare yet clinically significant late complication. While arachnoid cysts are typically regarded as congenital anomalies, several case reports and retrospective studies have identified instances of acquired cystic lesions developing months to years post-aSAH. In conclusion, the formation of arachnoid cysts following aSAH represents a clinically underrecognized phenomenon with potentially profound neurological implications.

## Data Availability

The original contributions presented in the study are included in the article/Supplementary Material, further inquiries can be directed to the corresponding authors.
